# The AISP Network of cross-sector data linkage efforts: Results & lessons from a national survey of 40 state and local systems in the US

**DOI:** 10.23889/ijpds.v9i5.2829

**Published:** 2024-09-18

**Authors:** Emily Berkowitz, Della Jenkins, Amy Hawn Nelson, Sharon Zanti, Deja Kemp, TC Burnett, Jessie Rios Benitez

**Affiliations:** 1 University of Pennsylvania

## Objective and Approach

This session will share findings from a national survey of 40 state and local cross-sector data linkage efforts using data to impact policy and outcomes across the United States (US). The survey, conducted in February 2023 by Actionable Intelligence for Social Policy (AISP) at the University of Pennsylvania, captured each effort’s purpose(s) for sharing data (the “why”), approach to linkage (the “how”), and the data sources being integrated (the “what”). Respondents are all current or prospective members of the AISP network, representing a cross-section of systems at different stages of development and engaged in peer exchange.

## Results

The presentation will share results that describe the core purposes of system development and how systems operate; this includes governance structures to institutionalize sharing relationships; staffing, funding sources, and operating budgets; legal frameworks; and technical infrastructure. The presentation will also explore what data systems share, and the role a peer-led community of practice plays in developing and honing approaches to public sector data use.

## Conclusions

Network members have demonstrated widespread impact in their communities. In the last two years, respondents collectively reported that their integrated data enabled 365 reports and briefings for policymakers to affect policy change and 160 research articles to advance knowledge for impact. They also widely reported improved relationships and trust across agencies and improved ability to evaluate what works.

## Implications

This presentation showcases the impact of the US community of practice in supporting diverse models for impact-oriented data systems.

